# Application of machine learning and artificial intelligence in the diagnosis and classification of polycystic ovarian syndrome: a systematic review

**DOI:** 10.3389/fendo.2023.1106625

**Published:** 2023-09-18

**Authors:** Francisco J. Barrera, Ethan D.L. Brown, Amanda Rojo, Javier Obeso, Hiram Plata, Eddy P. Lincango, Nancy Terry, René Rodríguez-Gutiérrez, Janet E. Hall, Skand Shekhar

**Affiliations:** ^1^ Department of Epidemiology, Harvard TH Chan School of Public Health, Boston, MA, United States; ^2^ Plataforma INVEST Medicina, Universidad Autónoma de Nuevo León- Knowledge Education Research (UANL-KER), Unit Mayo Clinic (KER Unit Mexico), Universidad Autónoma de Nuevo León, Monterrey, Mexico; ^3^ Reproductive Physiology and Pathophysiology Group, Clinical Research Branch, National Institute of Environmental Health Sciences, National Institutes of Health, Research Triangle Park, NC, United States; ^4^ Knowledge and Evaluation Research Unit-Endocrinology (KER-Endo), Mayo Clinic, Rochester, MN, United States; ^5^ Division of Library Services, Office of Research Services, National Institutes of Health, Bethesda, MD, United States; ^6^ Endocrinology Division, Department of Internal Medicine, University Hospital “Dr. José E. González”, Universidad Autonoma de Nuevo Leon, Monterrey, Mexico

**Keywords:** artificial intelligence, machine learning, polycystic ovarian syndrome (PCOS), diagnosis, classification, Stein-Leventhal syndrome

## Abstract

**Introduction:**

Polycystic Ovarian Syndrome (PCOS) is the most common endocrinopathy in women of reproductive age and remains widely underdiagnosed leading to significant morbidity. Artificial intelligence (AI) and machine learning (ML) hold promise in improving diagnostics. Thus, we performed a systematic review of literature to identify the utility of AI/ML in the diagnosis or classification of PCOS.

**Methods:**

We applied a search strategy using the following databases MEDLINE, Embase, the Cochrane Central Register of Controlled Trials, the Web of Science, and the IEEE Xplore Digital Library using relevant keywords. Eligible studies were identified, and results were extracted for their synthesis from inception until January 1, 2022.

**Results:**

135 studies were screened and ultimately, 31 studies were included in this study. Data sources used by the AI/ML interventions included clinical data, electronic health records, and genetic and proteomic data. Ten studies (32%) employed standardized criteria (NIH, Rotterdam, or Revised International PCOS classification), while 17 (55%) used clinical information with/without imaging. The most common AI techniques employed were support vector machine (42% studies), K-nearest neighbor (26%), and regression models (23%) were the commonest AI/ML. Receiver operating curves (ROC) were employed to compare AI/ML with clinical diagnosis. Area under the ROC ranged from 73% to 100% (n=7 studies), diagnostic accuracy from 89% to 100% (n=4 studies), sensitivity from 41% to 100% (n=10 studies), specificity from 75% to 100% (n=10 studies), positive predictive value (PPV) from 68% to 95% (n=4 studies), and negative predictive value (NPV) from 94% to 99% (n=2 studies).

**Conclusion:**

Artificial intelligence and machine learning provide a high diagnostic and classification performance in detecting PCOS, thereby providing an avenue for early diagnosis of this disorder. However, AI-based studies should use standardized PCOS diagnostic criteria to enhance the clinical applicability of AI/ML in PCOS and improve adherence to methodological and reporting guidelines for maximum diagnostic utility.

**Systematic review registration:**

https://www.crd.york.ac.uk/prospero/, identifier CRD42022295287.

## Introduction

Polycystic Ovary Syndrome (PCOS) is the most common endocrinopathy in reproductive aged women, with an estimated prevalence ranging from 4% to 20% and affecting more than 66 million worldwide in 2019 (1–5). PCOS is associated with increased incidence of cardiovascular disease, infertility, and of endometrial cancer (6–9). Its public health burden is immense, with nearly eight billion US dollars spent in 2020 to manage PCOS-related symptoms among women in the United States alone (10).

The diagnosis of PCOS is based on clinical criteria, with the Rotterdam criteria/International PCOS criteria (11, 12) being the most widely accepted. PCOS is characterized by the presence of a combination of hyperandrogenism, ovulatory dysregulation, and polycystic ovarian morphology (PCOM) (13–15). This already heterogenous clinical phenotype is complicated further by the elaborate interplay of genetic and environmental factors, such as diet related obesity or lifestyle factors, which affect clinical presentation (16). The criteria-based diagnosis of PCOS is complicated by variations in the clinical assessment of hyperandrogenism and determination of menstrual irregularity. Furthermore, the variation in normative standards for PCOM compounds these challenges (17). Estimates suggest that diagnosis is delayed by more than two years in one third of women with PCOS; yet this is likely an underestimation (18).

Artificial intelligence (AI) refers to simulation of human intelligence by computer based systems (19). On the other hand, machine learning (ML) is a subdivision of AI focused on learning from previous events and applying this knowledge to future decision making (20). ML techniques can be sub-classified as either supervised or unsupervised (21). The revolutionary advances in AI and ML over the last decade promise to rapidly advance our ability to diagnose and manage PCOS. This is in part due to the ability of AI to process massive amounts of disparate data, making it an ideal aid in the diagnosis of heterogeneous disorders like PCOS.

Several studies have investigated the ability of ML models to synthesize such disparate data as family genetic history, biomarkers, and demographic information into a unified algorithm for the diagnosis of PCOS, and make diagnostic predictions (22). Some pitfalls of these studies are their small size (22), lack of relevant comparators (23), use of varied diagnostic criteria (24, 25), and heterogeneity in reporting structures. Thus, the real gaps in knowledge and the full scope of AI/ML in the diagnosis of PCOS remain unclear. To better understand and summarize the body of evidence related to the application of AI/ML in PCOS, we conducted a systematic review of all relevant studies published up to January 1, 2022.

## Methods

### Study overview and eligibility criteria

This manuscript employed the Preferred Reporting Items for Systematic Reviews and Meta-analysis (PRISMA) guidelines, and was submitted to PROSPERO (record number PROSPERO 2022 CRD42022295287) (26). We included English language, peer-reviewed original studies that evaluated the use of AI/ML in diagnosing, classifying, stratifying, or predicting PCOS. We subdivided studies into those that ‘diagnosed’ and those that ‘classified’ PCOS subjects. Studies were considered to diagnose PCOS if they employed standard diagnostic criteria such as NIH, Rotterdam, androgen excess-PCOS and international PCOS criteria. In contrast, those studies that partially used standard criteria or only used some measures to determine PCOS were considered to ‘classify’ subjects as having PCOS.

### Data sources and search strategy

We applied a search strategy developed in collaboration with an experienced librarian to find potentially eligible studies. Databases searched were MEDLINE, Embase, the Cochrane Central Register of Controlled Trials, the Web of Science, and the IEEE Xplore Digital Library. The search included all articles from the time of inception of the dataset to May 2019. Conference abstracts were included if they fulfilled the eligibility criteria provided the manuscript wasn’t published. The full search strategy is included in **Supplementary Material 3**.

### Study selection

We uploaded all references to Covidence and performed two rounds of screening, title-and-abstract screening, and full-text screening. Each article was assessed for eligibility by two independent reviewers in both rounds of screening using standardized instructions. Pilot phases were conducted before each screening round to ensure a baseline understanding of the eligibility criteria and resolve misunderstandings between reviewers. Inter-rater reliability assessed through Cohen’s Kappa statistic was high (κ>0.80) in both rounds of screening.

In the first screening round, disagreements were included in the second round. In the second round, disagreements were resolved by consensus between reviewers or by arbitration of a third trained reviewer.

### Data collection and management

Five reviewers working independently and in duplicate extracted data from studies using a standardized extraction form. Two pilot phases were performed to ensure proficiency in the data extraction procedure. Further disagreements were discussed and resolved by consensus, and the database was cleaned by two reviewers. The extracted variables were: 1) study characteristics (authors’ information, publication year, country and setting, study design, aim and type of machine learning used, and type of data entered into the models); 2) artificial intelligence/machine learning characteristics (type of dataset used, dataset independence, type of results reported [sensitivity, specificity, area under the curve, diagnostic accuracy, precision]); 3) PCOS characteristics (definition of the disease, sample size); and 4) risk of bias.

### Risk of bias

Each study was assessed for risk of bias by two independent reviewers and disagreements were resolved by two separate reviewers. We used a modified version of the Quality Assessment of Diagnostic Accuracy Studies (QUADAS-2) tool, which includes four domains: patient selection, index test, reference standard, and flow and timing. As this tool is not designed for systematic reviews of diagnostic accuracy studies using AI/ML interventions, we summarized and complemented it with input from the authors to ensure that critical questions for AI/ML interventions were included in addition to the relevant pre-existing QUADAS-2 questions. Details of the modified QUADAS-2 tool are provided in **Supplementary Material 2**. The tailored QUADAS-2 tool was piloted on five studies by all reviewers and differences resolved with consensus. If a study had at least two domains at unclear risk of bias without any domain deemed at high risk of bias, it was judged to be at unclear risk of bias. Finally, studies with domains classified as low risk of bias without any domain of unclear or high risk of bias were considered low risk of bias.

## Results

### Characteristics of the included studies

**Figure 1:**
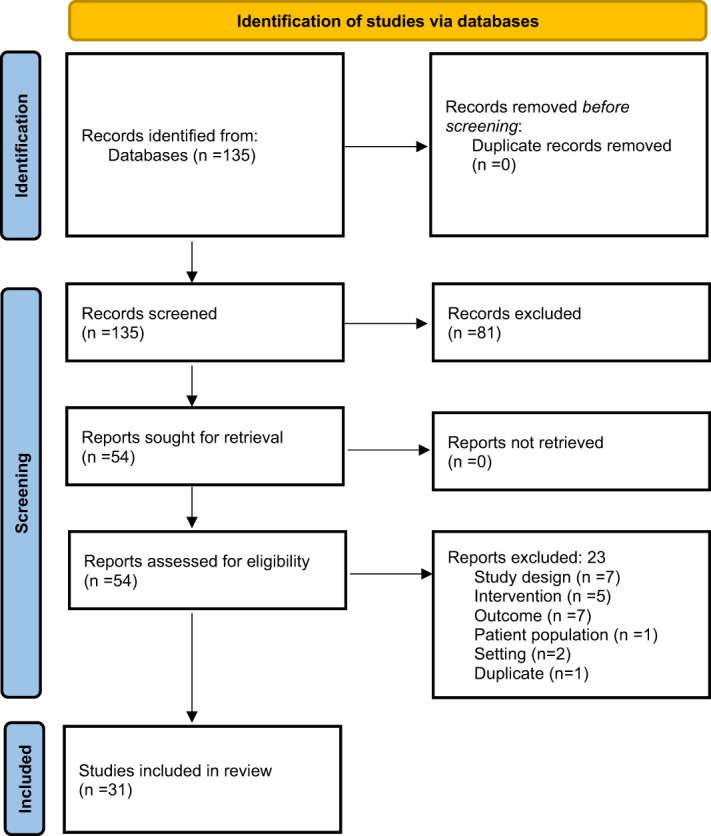
Selection process of the studies. Article selection flow chart for studies related to AI/ML and PCOS according to Preferred Reporting Items for Systematic Reviews and Meta-Analyses (PRISMA) guidelines.

A total of 31 studies met our inclusion criteria (**Figure 1**). All studies were observational and used retrospective data samples to assess the performance of the AI/ML process on the diagnosis or classification of patients. Seven of 31 studies (23%) were multi-center studies and many were conducted either in India (29%) or in China (16%). Eleven studies (36%) included subjects who did not have PCOS as controls. Sample size ranged from 9 to 2,000 patients with PCOS and the median age of participants included in studies was 29 years. The rest of the general characteristics can be found in **Table 1**.

**Table 1:** Characteristics of the included studies.

	Author, year	Multi-center	Country	Study type (interventional vs. experimental)	Controls	Type of data	Subtype of data	Definition of cases	N	Age	Definition of controls	N	Age	Aim	Comparator
1	Nazarudin et al., 2020 (27)	No	Malaysia	Observational	No	Imaging	Ultrasound	PCOS ultrasound images	13	NR	–	–	–	Classification	None
2	Bharati et al., 2020 (28)	Yes	Bangladesh	Observational	Yes	Clinical, and imaging data	Anthropometric and hormonal features; Ultrasound	Clinical diagnosis	117	NR	Clinical diagnosis	364	NR	Diagnosis	None
3	Cahyono et al., 2017 (29)	No	Indonesia	Observational	Yes	Imaging	Ultrasound	NR	14	NR	NR	40	NR	Classification	None
4	Castro et al., 2015 (30)	Yes	USA	Observational	Yes	Electronic medical records	Signs, symptoms, comorbidities, medication, laboratory results, ultrasound findings	ICD-9 code 256.4	NR	NR	NR	NR	NR	Diagnosis	ICD-9 codes
5	RoyChoudhury et al., 2016 (31)	No	India	Observational	Yes	Metabolomics	Aminoacids and energy metabolites	Rotterdam Criteria	68	28.75 ± 4.28	Age-matched healthy non-PCOS women undergoing tubal ligation	74	29.65 ± 3.69	Classification	None
6	Rodriguez et al., 2020 (32)	No	USA	Observational	–	Virtually generated clinical data	Signs and symptoms	Rotterdam Criteria	9	NR	NR	NR	NR	Screening/classification	Board-certified reproductive endocrinology and infertility physician
7	Purnama et al., 2015 (33)	No	Indonesia	Observational	Yes	Imaging	Ultrasound	NR	20	NR	NR	60	NR	Classification	None
8	Prapty et al., 2020 (34)	Yes	Bangladesh	Observational	Yes	Clinical data	Antropometric, hormonal, and menstrual cycle data	NR	NR	NR	NR	NR	NR	Diagnosis	None
9	Chauhan et al., 2021 (35)	No	India	Observational	Yes	Clinical data	Symptoms and menstrual cycle data	Women with PCOS	61	>18	normal non-PCOS cases	206	>18	Screening/classification	None
10	Lawrence et al., 2007 (36)	No	Canada	Observational	Yes	Imaging	Ultrasound	Polycystic ovaries	33	NR	normal ovaries	37	NR	Classification	None
11	Mehrotra et al. et al., 2011 (23)	No	India	Observational	Yes	Clinical data	Menstrual cycle, metabolic and clinical data	Clinical criteria*	150	31.24	NR	100	32.24 ± 2.02	Diagnosis	None
12	Matharoo-Ball et al., 2007 (37)	No	U.K	Observational	Yes	Proteomics	Serum proteins/peptide biomarkers	Rotterdam Criteria	12	NR	age- and BMI-matched control	12	NR	Classification	None
13	Lehtinen et al., 1997 (38)	No	Finland	Observational	Yes	Clinical data	Hormones and blood biomarkers	Adams criteria	54	27 ± 6 (14-38)	regularly menstruating volunteers with normal ovaries	29	33 ± 5 (23-41)	Classification	None
14	Kumar, et al., 2014 REFID 101 (39)	No	Bangalore	Observational	Yes	Imaging	Ultrasound images	Anovulatory infertily/PCOS	210 Images	25-35	Normal	210 Images	25-35	Classification	None
15	Madhumitha et al., 2021 (40)	No	India	Observational	No	Imaging	Ultrasound	NR	NR	NR	NR	NR	NR	Classification	Physical identification
16	Ho et al., 2020 (41)	Yes	Taiwan	Observational	Yes	Genetics	Gene expression microarray	2009 Rotterdam Criteria and 1990 NIH criteria	48	NR	normal ovulatory women without hyperandrogenism	181	NR	Classification	None
17	Gopalakrishnan et al., 2021 (42)	No	India	Observational	Yes	Imaging	Ultrasound	PCOS imaging	35	NR	Normal Imaging of ovary	55 (30 normal + 25 cystic)	NR	Classification	None
18	Dong et al., 2015 (43)	No	China	Observational	Yes	Clinical data	Lipids, amino acids, carbohydrates, organic acids, nucleosides and aliphatic acyclic compounds	2003 Rotterdam criteria	20	25.1 ± 4.51	Normal menstrual cycle, none clinical and biochemical hyperandrogenism	15	26.4 ± 2.92	Classification	None
19	Deshpande et al., 2014 (44)	No	India	Observational	Yes	Clinical and imaging	Ultrasound, hormones and clinical data	NIH criteria	9	NR	NR	20	NR	Diagnosis	Manual detection and physician verification
20	Denny et al., 2019 (45)	Yes	India	Observational	Yes	Clinical data and imaging	Ultrasound, physiological symptoms, biochemical data	NR	177	18 to 40	Normal or Non-PCOS	364	18 to 40	Diagnosis	None
21	Deng et al., 2011 (46)	No	China	Observational	No	Imaging	Ultrasound	PCOS imaging	31	NR	NR	NR	NR	Classification	None
22	Dapas et al., 2020 (47)	Yes	USA	Observational	Yes	Genome wide association	Biochemical and genotype	NIH criteria	893	28 (25–32) median, IQR	phenotyped reproductively normal control women	4098	NR	Classification	None
23	Che et al., 2019 (48)	No	China	Observational	Yes	Genetics	Aberrant circular RNA (circRNA) expression profiles	Rotterdam revised criteria	20	NR	Who underwent IVF treatment for an indication of male factor infertility	20	NR	Classification	None
24	Cheng et al., 2019 (49)	No	USA	Observational	No	Imaging	Ultrasound	2003 Rotterdam criteria	2000	31.4	NR	NR	NR	Classification	None
25	Zhang et al, 2021 (50)	No	China	Observational	No	Clinical data	Metabolic data	Rotterdam Criteria	50	30.24 ± 3.24	Regular menstrual cycles and normal ovarian reserve who sought treatment for infertility due to a tubal or male factor	NR	NR	Classification	None
26	Xie et al, 2020 (51)	Yes	Denmark, Ireland, India, China, USA, UK	Observational	No	Genetics	Gene expression microarray	NR	76	NR	NR	NR	57	Classification	None
27	Thakre et al, 2020 (52)	No	India	Observational	No	Clinical data	Physical and medical parameters, along with physical symptoms	NR	177	32	NR	364	31	Classification	None
28	Vikas et al, 2018 (53)	No	India	Observational	No	Clinical data	Lifestyle and food habits	NR	119	18-22	NR	NR	NR	Diagnosis	None
29	Setiawati, et al., 2016 (54)	No	Indonesia	Observational	No	Imaging	Ultrasound images	NR	2	NR	NR	NR	NR	Classification	None
30	Rihana et al, 2013 (55)	No	Lebanon	Observational	Yes	Imaging	Ultrasound images	NR	20	NR	Healthy non-containing cysts	20	NR	Classification	None
31	Deng et al, 2008 (56)	No	China	Observational	No	Imaging	Ultrasound images	NR	NR	NR	NR	NR	NR	Classification	Manual image reading

Studies presented by lead author and year of publication with corresponding study characteristics. Age presented as median ± standard deviation when applicable. Shorthand denoted as: No Response (NR), Inner Quartile Range (IQR).

*The diagnosis of PCOS was made based on the following criteria: (1) Cycle length (oligomenorrhea) (2) clinical and metabolic features (3) polycystic ovarian morphology (presence of 12 or more follicles measuring 2-9 mm in diameter or increased ovarian volume) with the exclusion of other etiologies.

Nearly half of all studies (48%) used ultrasound images to implement the AI/ML intervention. Twelve studies (39%) used clinical data such as anthropometric features (10%), signs and symptoms (16%), biomarkers (19%), genetics (13%) and metabolomics or proteomics (10%).

Ten (32%) studies used a validated diagnostic criterion to select the population, such as exclusively the Rotterdam criteria (23%), the NIH Criteria (6%), with one study using a combination of NIH and Rotterdam criteria (3%) (11, 12, 57) (**Table 1**). Another study (3%) used the Adams criteria, an imaging-based criteria which has not been clinically validated (58). The remaining 20 studies (65%) used clinical information to make the diagnosis, with or without complementary imaging (55%), with one study using ICD codes. Two (7%) studies reported that they used age-matched participants without the diagnosis of PCOS as controls, while other studies reported scarce information about controls; including definitions such as “normal ovaries through imaging”, or “normal ovulation cycles”. Five (16%) studies provided no definition for controls.

### AI/ML models performance

**Figure 2:**
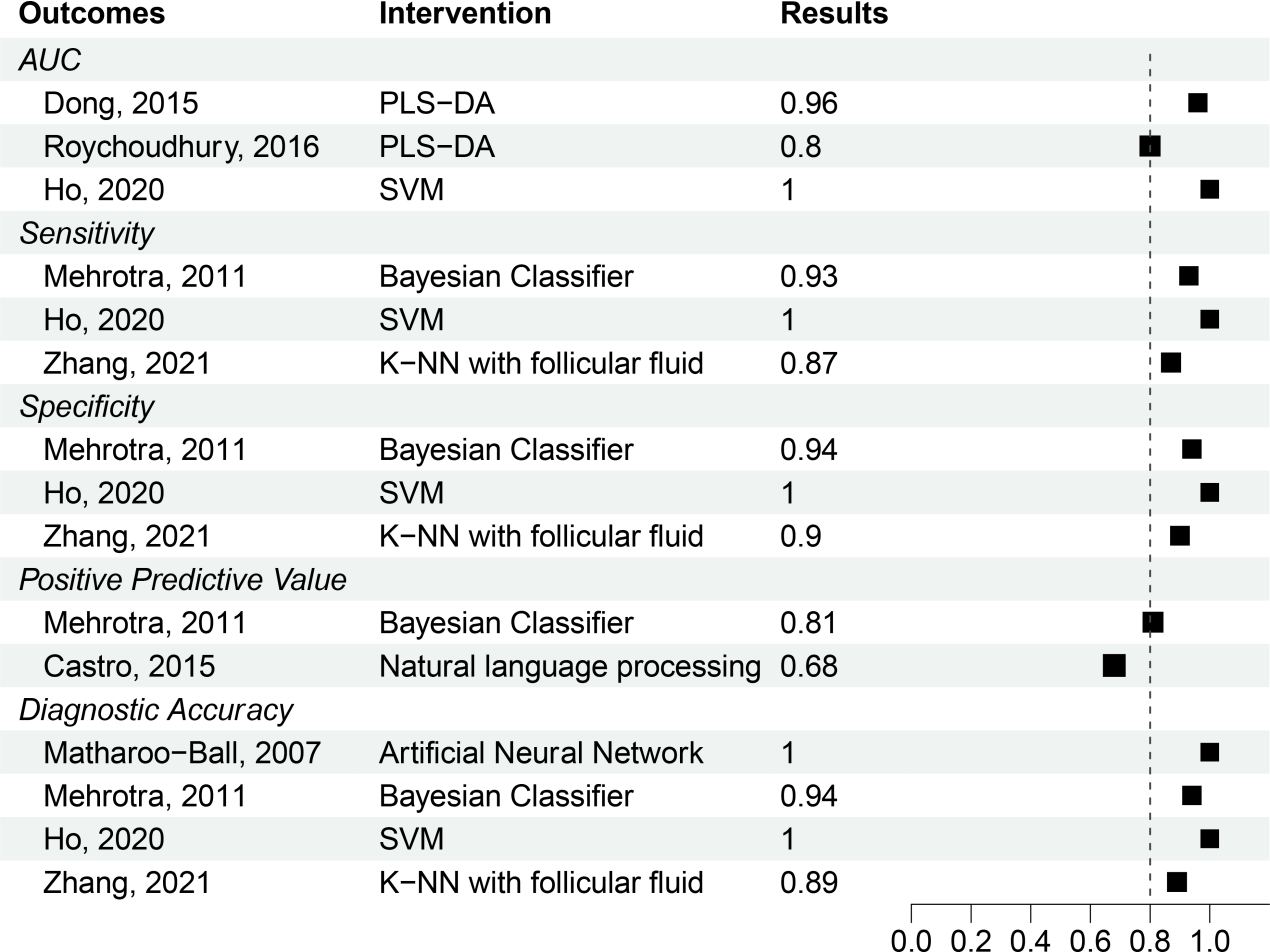
Unpooled results of studies with well-defined PCOS patient population. Outcomes and interventions are denoted in shorthand as Area Under the Curve (AUC), Partial Least-Squares Discriminant Analysis (PLS-DA), Support Vector Machine (SVM), and K-Nearest Neighbor (K-NN). A parameter threshold of 80% (0.8) indicated by the dotted line was considered a benchmark to evaluate studies assuming a 20% performance error.

Among the ten (32%) studies that used standardized diagnostic criteria, the area under the receiver operator curve ranged from 80% to 100% (n=3 studies), diagnostic accuracy from 89% to 100% (n=4 studies), sensitivity from 87% to 100% (n=3 studies), specificity from 90% to 100% (n=3 studies), and positive predictive value from 68% to 81% (n=2 studies), and negative predictive value (NPV) from 94% to 99% (n=2 studies). Performance measures for all the included studies are shown in **Table 2**. The studies that used standardized PCOS criteria are summarized in **Figure 2** by outcome type.

**Table 2:** Main findings of the included studies.

	Author	Type of data	AI/ML intervention	Best model	AUC	Sens	Spec	PPV	NPV	Diag. Acc.
1	Nazarudin, et al. (27)	Imaging	2 automated segmentation models: combination of Otsu’s thresholding and the Chan - Vese method, Otsu’s thresholding.	Chan-Vese + Otsu’s segmentation analysis	NR	NR	NR	NR	NR	Remarkable increase in accuracy
2	Bharati, et al. (28)	Clinical, and imaging data	Gradient boosting, RF, LR, and LR	Hybrid RFLR	0.93	NR	NR	NR	NR	0.91
3	Cahyono, et al. (29)	Imaging	Convolutional Neural Network	CNN	NR	NR	NR	NR	NR	
4	Castro, et al. (30)	Electronic medical records	Algorithm using Natural language processing and codified data	Algorithm using Natural language processing and codified data	NR	NR	NR	0.68	NR	NR
5	RoyChoudhury, et al. (31)	Metabolomics	PLS-DA	Statistical analysis with PLS-DA	0.8	NR	NR	NR	NR	NR
6	Rodriguez, et al. (32)	Virtually generated clinical data	Bayesian network	Bayesian network	NR	NR	NR	NR	NR	NR
7	Purnama, et al. (33)	Imaging	Neural Network - LVQ method, K-NN and SVM	SVM	NR	NR	NR	NR	NR	0.83
8	Prapty, et al. (34)	Clinical data	KNN, SVM, Naive Classifier, RF	RF	NR	NR	NR	NR	NR	0.94
9	Chauhan, et al. (35)	Clinical data	KNN, Naïve Bayes Classifier, SVM, Decision tree classifier, LR	Decision Tree Classifier	NR	0.41	0.94	NR	NR	0.81
10	Lawrence, et al. (36)	Imaging	LDC, KNN, SVM	LDC	NR	0.91	0.95	NR	NR	0.93
11	Mehrotra, et al. (23)	Clinical data	Multivariate logistic regression, Bayesian Classifier	Bayesian classifier	NR	0.93	0.94	0.81	NR	0.94
12	Matharoo-Ball, et al. (37)	Proteomics	Artificial Neural Network	Artificial Neural Network	NR	NR	NR	NR	NR	1
13	Lehtinen, et al. (38)	Clinical data	TPFFN and SOM	TPFFN	NR	NR	NR	NR	NR	efficiency of 97%
14	Kumar, et al., 2014 REFID 101 (39)	Imaging	PNN, SVM, RBF	PNN	NR	NR	NR	NR	NR	0.98
15	Madhumitha, et al. (40)	Imaging	SVM, K-NN, LR	Proposed Method (SVM + K-NN + LR)	NR	NR	NR	NR	NR	0.98
16	Ho, et al. (41)	Genetics	SVM, RF, GMM	SVM with 5 and 3-fold cross validation	1	1	1	NR	NR	1
17	Gopalakrishnan, et al. (42)	Imaging	SVM.	SVM	NR	NR	NR	NR	NR	0.94
18	Dong, et al. (43)	Clinical data	Orthogonal PLS-DA	Orthogonal PLS-DA	0.96	NR	NR	NR	NR	NR
19	Deshpande, et al. (44)	Clinical and imaging	SVM	SVM	NR	NR	NR	NR	NR	0.95
20	Denny, et al. (45)	Clinical data and imaging	LR, KNN, CART, RFC, NB, SVM	RFC	NR	0.74	0.98	NR	NR	0.89
21	Deng, et al. (46)	Imaging	Watershed + Object growing algorithm, Level set method, boundary vector field methiod, fuzzy support vector machine classifier	Watershed + Object growing algorithm	NR	NR	NR	NR	NR	NR
22	Dapas, et al. (47)	Genome wide association	SVM, RF, GMM	NR	NR	NR	NR	NR	NR	NR
23	Che, et al. (48)	Genetics	Unsupervised hierarchical clustering analysis	Unsupervised hierarchical clustering analysis	NR	NR	NR	NR	NR	NR
24	Cheng, et al. (49)	Imaging	Gradient boosted trees, Rules based classifier	Rules-based classifier	NA	0.97	0.98	0.95	0.99	0.98
25	Zhang, et al. (50)	Clinical data	K-NN, RF, XGB, Stacking classification model	K-NN with follicular fluid	NR	0.87	0.90	NR	NR	0.88
26	Xie, et al. (51)	Genetics	Random Forest, Artificial Neural Network	Artificial Neural Network	0.73	0.73	0.75	NR	NR	NR
27	Thakre, et al. (52)	Clinical data	RF, SVM, LR, Gaussian Naïve Bayes, K-NN	RFC	0.89	0.97	0.8	0.89	0.94	0.91
28	Vikas, et al. (53)	Clinical data	Frequent item set mining, Apriori algorithm	NR	NR	NR	NR	NR	NR	NR
29	Setiawati, et al. (54)	Imaging	LR, SVM, Backpropagation Neural Network	Backpropagation Neural Network	NR	NR	NR	NR	NR	NR
30	Rihana, et al. (55)	Imaging	SVM	SVM	NR	0.88	0.95	NR	NR	0.9
31	Deng, et al. (56)	Imaging	Clustering analysis, Manual image reading	Clustering analysis	0.84	NR	NR	NR	NR	0.84

Studies presented by lead author and year of publication with corresponding main findings. Shorthand denoted as: No Response (NR), K-Nearest Neighbor (K-NN), learning vector quantization (LVQ), logistic regression (LR), not reported (NR), support vector machine (SVM), partial least squares discriminant analysis (PLS-DA), topology-preserving feed-forward network (TPFFN), extreme gradient boosting (XGB), self-organizing map (SOM). Classification and Regression Trees (CART), Random Forest (RF), Random Forest Classifier (RFC), Naïve Bayes Classifier (NB), Gaussian mixed model (GMM), Linear Discriminant Classifier (LDC), Convolutional Neural Network (CNN), Random Forest and Logistic Regression (RFLR)

### Machine learning methods

The majority (71%) of studies we investigated used supervised methods. The most common were support vector machine (SVM) (42%), K-nearest neighbor (26%), regression models (23%), and Random Forest (23%). Unsupervised methods were used in nine (29%) studies and included neural networks (13%), Otsu’s thresholding and Watershed + object growing algorithm (6%), clustering analysis (6%), and self-organizing maps (3%) (**Table 2**). Various AI/ML models are described in **Table 3**.

**Table 3:** Machine Learning Methods.

Type of Machine Learning	Description of Technique
Unsupervised Learning	Hidden patters within unlabeled datasets are identified through clustering or association (C-means, K-means, etc)
Reinforcement Learning	Sequential feedback is provided to models based on their response to training data (Q-learning, SARSA, etc)
Semi-Supervised Learning	Models are trained with a small amount of initial data before being used to identify structures within larger unlabeled datasets (Generative model, semi-supervised SVM, etc).
Supervised Learning	Labeled inputs and outputs are used to approximate a relationship between variables (ie linear regression, logistic regression, SVM, KNN, etc).

Definitions of Machine Learning Techniques and Sample Methods. Techniques are shortened to SARSA (State, Action, Reward, State, Action), SVM (Support Vector Machine), and KNN (K Nearest Neighbor).

Only six (19%) studies performed all major steps of training, testing, and validation in their AI/ML interventions. About three-quarters of studies (74%) performed at least one of these steps. Specifically, ten (32%) studies performed only training and testing, four (13%) only training and validation, and three (10%) completed only one of them. Among those studies that used at least two steps, all used an independent data set for each step by using a proportion of their sample for each step or cross-validation models (where data is trained and tested on different observations).

Nineteen (61%) studies compared the effectiveness of two or more AI/ML interventions on the same sample, while only three (10%) compared AI/ML interventions against a non-machine learning classifier (board-certified physician or ICD-9 codes). Of these three, two studies described the criteria used by the clinician or the codes used to make the diagnosis.

### Risk of bias

Overall, the risk of bias was judged to be high across all studies (**Supplementary Material 1**). Six (19%) studies described using a consecutive or random sample of the enrolled patients. Moreover, five (16%) studies used validated criteria to select their population, which affected risk of bias due to misclassification bias but also applicability bias due to an unclearly defined patient population in the studies. About half of all (52%) studies used an independent dataset to validate the AI/ML intervention. Finally, nine (29%) studies had hospital affiliations or a physician as a co-author of the study.

## Discussion

We performed a systematic review of AI/ML interventions in PCOS. All included studies were observational and retrospective. A small number used standard inclusion criteria such as the NIH, Rotterdam, or International PCOS criteria for diagnosis. Most studies achieved a high ability to diagnose PCOS or ‘classify’ patients as having PCOS using AI informed by clinical, radiological, electronic health records or biochemical data. Among the ten studies that used standardized criteria, the area under the receiver operator curve ranged from 80% to 100%, diagnostic accuracy from 89% to 100%, sensitivity from 87% to 100%, specificity from 90% to 100%, and positive predictive value from 68% to 81%. The most common AI/ML methods were SVM in 13 (42%) studies, K-nearest neighbor in eight (26%) studies, and regression models in seven (23%) studies. Importantly, a large number of the studies analyzed in the current review were able to achieve a high degree of diagnostic accuracy relative to standardized criteria. For instance, Deshpande et al. (2014) attained a 95% diagnostic accuracy against the Rotterdam criteria using an SVM algorithm using ultrasound imaging, clinical, and biochemical data (44). Similarly, Bharti et al. (2020) employed multiple ML algorithms to a dataset of 364 women with and without PCOS using clinical and imaging data and reported a > 90% diagnostic accuracy for the best SVM model (28).

AI/ML-based screening techniques for diabetic retinopathy and colorectal cancer have previously been found to be highly cost-effective (59, 60). In the case of colorectal cancer, cost savings of 400 million USD have been estimated when comparing next generation sequencing approaches to AI-based screening techniques (61). The potential use of AI/ML in the diagnosis and management of endocrine disorders has sparked intense research activity. A recent review reported that among the 611 ML-based endocrinology studies published between 2015 and 2020, 52% focused on diabetes, 14% on retinopathy, 14% on thyroid dysfunction, 8% on endocrine-related carcinoma, 7% on osteoporosis, and 5% on other disease states (62). Despite a growth in such studies, FDA-approved applications of AI for diagnostic or therapeutic purposes have lagged and approved devices employing AI/ML are concentrated in the management of diabetes and related conditions (63, 64).

In comparison, polycystic ovarian syndrome represents an ideal setting for future AI-based tools, given its high prevalence, significant healthcare burden, delayed detection, and complex diagnostic criteria spanning clinical, biochemical, and radiological domains. The diagnostic delay of greater than two years in a third of women reporting PCOS symptoms is a potent target for AI/ML-based approaches (18). Furthermore, geographical heterogeneity in clinical features of PCOS suggests an additional role of environmental influences, which may be overcome through adoption of AI/ML (65). Together, high costs and diagnostic delays in PCOS present a major unmet need which could be filled by the adoption of AI technology, as effectively demonstrated in other diseases. AI holds especially high potential for the diagnosis of PCOS because of its heterogeneous nature, with clinical, biochemical and radiological features each being incorporated into its diagnostic criteria (12). The use of AI on electronic health record (EHR) systems holds the potential to integrate these features while reducing diagnostic delays in PCOS.

The current body of research on AI in PCOS has revealed high rates of sensitivity and accuracy of PCOS detection. This implies that a well-designed AI/ML based program has the potential to significantly enhance our capability to diagnose PCOS early, with associated cost savings and a reduced burden of PCOS on patients and on the health system. However, several gaps remain in the domain of AI/ML based detection of PCOS. First, we noted that only a third of studies (32%) used standardized criteria such as the Rotterdam, NIH and International PCOS criteria as reference standards when evaluating AI in PCOS. This presents a high possibility misclassification of disease and biased detection estimates. Second, there was considerable heterogeneity in assessed AI-based studies, with some relying exclusively on a single parameter of PCOS diagnosis such as radiological, biochemical, or clinical features, despite Rotterdam criteria recommending diagnosis based on more than one of these elements. Third, a large number of assessed studies did not exhaustively report methodology/algorithms for AI based diagnosis, presenting concerns about the reproducibility of their findings. Most studies also relied on observational/retrospective data without use of prospective studies or validation datasets, limiting their applicability (66). A fourth major gap was the inadequate utilization of electronic health records, one of the most promising avenues for AI integration due to their potential for synthesizing clinical, biochemical, radiological, and genetic information and reducing lead time to the diagnosis in PCOS. This warrants further investigation in future studies. Finally, we noted that a vast number of AI/ML based studies were conducted in non-healthcare settings (71%) with non-healthcare investigators (97%). This raises the possibility of reduced applicability and relevance of studies in the clinical management of PCOS since such studies, while being technically robust, may not account for clinically important variables and outcomes. It is therefore important for physicians to become more aware of the advantages of AI/ML based methodologies and for physicians and computational scientists interested in AI/ML to work together to optimize the power of these new tools. Moreover, future AI/ML studies with applications for PCOS or other conditions, should make greater efforts to increase the methodological quality to increase the validity of the results. For this, we recommend the following five measures to improve the applicability of AI/ML for diagnosing PCOS and improving its care.

1. Increase collaboration between clinicians, researchers, and computational biologists.

2. Set up combined registries of data that include defined clinical, radiological (including images), and laboratory data (with reference values) of PCOS patients.

3. Use standardized criteria to train machine learning models as the standard reference and perform robust training and validation studies in PCOS patients.

3. Since some of the data used to develop the model may have some variation by time, it is important that future studies also test for performance (accuracy measures) consistency across time.

4. Enhance integration of population-based studies [e.g. *All of Us, NHANES* (67, 68)] with electronic health datasets to identify risk factors and risk enhancers for PCOS.

5. Include commonly used biochemical tests such as AMH, gonadal hormones, markers of insulin resistance and others in AI/ML to identify reliable biomarkers that can aid the diagnosis of PCOS.

To our knowledge, this is the first systematic review of AI/ML in the diagnosis of PCOS, spanning all published studies to date. We followed the methodological standards for systematic reviews proscribed by PRISMA guidelines. Despite the absence of a methodological assessment tool for evaluation of AI/ML based studies at the time of execution of this review, we performed a thorough evaluation of the quality by adapting the QUADAS-2 tool and adding relevant questions for the AI/ML interventions evaluated. Although not a weakness of our methods, confidence in our results is limited by the relatively small number of studies conducted on this subject, the heterogeneity of available data, and the risk of bias in primary studies. Broadly, poor dataset sourcing using non-standardized criteria, inconsistent use of best-practice machine learning methods, and limited clinical affiliations among authorship all undermined confidence in our selected studies.

In conclusion, our findings suggest that there is a high potential of AI/ML based programs in the diagnosis and care of PCOS, but that future studies should focus on enhancing methodological robustness and incorporating variables and outcomes of clinical importance.

## Data availability statement

The original contributions presented in the study are included in the article/**Supplementary Material**. Further inquiries can be directed to the corresponding author.

## Author contributions

FB and SS designed the study. FB and EB participated in all phases of the conduction of the study. FB, EB, SS, AR, JO, HP, and EL participated in screening, data extraction, and manuscript writing. JH, RR-G and SS reviewed the final version of the manuscript. All authors contributed to the article and approved the submitted version.
