# Supplementary material for: Application of machine learning and artificial intelligence in the diagnosis and classification of polycystic ovarian syndrome: a systematic review
Source: Front Endocrinol (Lausanne). 2023 Sep 18;14:1106625. doi: 10.3389/fendo.2023.1106625 (PMC10542899; doi:10.3389/fendo.2023.1106625)
Supplement: Supplementary file 1 [file Table_1.docx]

**Supplement Material 1. Risk of bias evaluation for all included studies.**

|  | **Study** | **Risk of bias** | | | | | | **Applicability Concerns** | | **Other** | | | | | | |
| --- | --- | --- | --- | --- | --- | --- | --- | --- | --- | --- | --- | --- | --- | --- | --- | --- |
|  |  | **D1** | | | | **D3** | | **D1** | **D3** |  |  |  |  |  |  |  |
|  |  | **Q1** | **Q2** | **Q3** | **Q4** | **Q5** | **Q6** | **Q7** | **Q8** | **Q9.1** | **Q9.2** | **Q10** | **Q11** | **Q12** | **H/C** | **MD** |
| 1 | Nazarudin 2020 | U | U | U | U | U | U | H | U | U | U | N | N | U | N | N |
| 2 | Bharati 2020 | N | N | Y | U | U | U | U | U | Y | Y | U | Y | U | N | N |
| 3 | Cahyono 2017 | U | N | U | U | U | U | U | U | Y | Y | U | N | U | N | N |
| 4 | Castro 2015 | Y | Y | Y | L | Y | L | L | L | Y | N | Y | Y | Y | Y | N |
| 5 | RoyChoudhury 2016 | U | N | N | H | NA | L | L | L | Y | Y | Y | Y | Y | N | N |
| 6 | Rodriguez 2020 | Y | Y | Y | L | Y | L | L | L | N | N | N | N | Y | Y | Y |
| 7 | Purnama 2015 | U | N | U | U | U | U | U | U | N | N | U | N | N | N | N |
| 8 | Prapty 2020 | U | N | U | U | U | U | U | U | Y | N | Y | N | U | N | N |
| 9 | Chauhan 2021 | Y | Y | U | H | N | H | H | H | Y | N | U | N | N | N | N |
| 10 | Lawrence 2007 | U | U | U | U | NA | U | U | U | Y | N | N | Y | U | Y | N |
| 11 | Mehrotra 2011 | Y | N | Y | L | Y | L | L | L | Y | N | N | Y | Y | N | N |
| 12 | Matharoo-Ball 2007 | U | N | U | U | NA | U | U | U | Y | Y | Y | Y | Y | N | N |
| 13 | Lehtinen 1997 | U | U | U | U | U | U | U | U | Y | N | N | N | N | Y | N |
| 14 | Kumar 2014 | U | U | U | U | NA | U | U | U | Y | Y | N | N | U | N | N |
| 15 | Madhumitha 2021 | U | U | Y | U | U | U | U | U | U | U | U | Y | U | N | N |
| 16 | Ho 2020 | Y | N | N | L | Y | L | L | L | U | U | N | Y | Y | Y | N |
| 17 | Gopalakrishnan 2021 | U | Y | U | U | N | U | U | U | Y | Y | N | Y | U | N | N |
| 18 | Dong 2015 | U | N | U | U | NA | L | L | L | N | N | N | Y | Y | Y | N |
| 19 | Deshpande 2014 | U | N | Y | U | U | U | U | U | U | U | N | U | U | N | N |
| 20 | Denny 2019 | U | U | U | U | U | U | U | U | Y | N | Y | Y | U | N | N |
| 21 | Deng 2011 | U | Y | U | U | NA | U | U | U | U | U | N | N | N | N | N |
| 22 | Dapas 2020 | U | Y | Y | U | NA | U | U | U | Y | N | Y | Y | Y | N | N |
| 23 | Che 2019 | N | N | Y | H | NA | L | L | L | U | U | N | Y | Y | Y | N |
| 24 | Cheng 2019 | Y | Y | N | L | NA | L | L | L | Y | N | N | Y | N | N | N |
| 25 | Zhang 2021 | U | N | Y | L | Y | L | L | L | U | U | N | Y | Y | Y | N |
| 26 | Xie 2020 | U | N | U | U | U | U | U | U | Y | N | Y | Y | N | Y | N |
| 27 | Thakre 2020 | U | U | U | U | U | U | U | U | U | U | U | N | U | N | N |
| 28 | Vikas 2017 | U | U | U | U | U | U | U | U | U | U | U | N | U | N | N |
| 29 | Setiawati 2016 | U | N | U | H | U | U | U | U | N | N | N | Y | U | N | N |
| 30 | Rihana 2013 | U | U | U | U | U | U | U | U | U | U | U | N | U | N | N |
| 31 | Deng 2008 | U | U | U | U | U | U | U | U | U | U | U | N | U | N | N |

Study evaluations were annotated with Y (Yes), N (No), U (Unclear), L (Low Risk), and H (High Risk), and NA (Not Applicable). Columns are annotated as Risk of Bias (ROB), Applicability Concerns (AC), Other (O), Doman 1: Patient Selection (D1), Domain 2: Index Test (D2), and Domain 3: Reference Standard (D3). Questions considered were the following. Q1: Was a consecutive or random sample of patients enrolled? Q2: Was a case-control design avoided? Q3: Did the study avoid inappropriate exclusions? Q4: Could the selection of patients have introduced bias? Q5: Is the reference standard likely to correctly classify the target condition? Q6: Could the reference standard, its conduct, or its interpretation have introduced bias? Q7: Is there concern that the included patients do not match the review question? Q8: Is there concern that the target condition as defined by the reference standard does not match the review question? Q9.1: Is the test dataset separate to the training dataset? Q9.2: Is the validation dataset separate to the training dataset? Q10: Is the dataset multi-centric? Q11: Was the method described in sufficient detail to reproduce the presented results? Q12: Was Rotterdam/NIH/ASRM criteria used? HC: Hospital/Clinic affiliation existed MD: Medical Doctor affiliation existed.
