# Supplementary material for: Application of machine learning and artificial intelligence in the diagnosis and classification of polycystic ovarian syndrome: a systematic review
Source: Front Endocrinol (Lausanne). 2023 Sep 18;14:1106625. doi: 10.3389/fendo.2023.1106625 (PMC10542899; doi:10.3389/fendo.2023.1106625)
Supplement: Supplementary file 2 [file Table_2.docx]

**Supplementary Material 2. About the Risk of Bias Tool: Modified QUADAS-2**

We excluded several questions because they were irrelevant for the assessment of risk of bias in our systematic review. All the decisions to modify the tool were in agreement between all the reviewers. These are discussed below.

**Risk of bias**

Domain 2. Index Test. Question: Were the index test results interpreted without knowledge of the results of the reference standard?

We decided to exclude this question because we believe that if the index test is a machine learning (ML) / artificial intelligence (AI) methodology, then, it wouldn’t matter if the results of the method are interpreted without the knowledge of the results of the reference standard because having this knowledge before reading the results of the index test could not affect the result of the index test and therefore could not introduce bias. This would be different to evaluating the result of an ultrasound imaging study after knowing what the laboratory results are for a particular patient. In this example, the knowledge about the laboratory results could influence the interpretation of the results of the imaginag study.

Domain 2. Index Test. Question: Could the conduct or interpretation of the index test have introduced bias?

We decided to exclude this question because we believe that the conduct or interpretation of the index test in this study including only ML/AI interventions would be independent of human manipulation or interpretation assuming this interventions are automated.

Domain 3. Reference Test. Question: Were the reference standard results interpreted without knowledge of the results of the index test?

We decided to exclude this question under the assumption that the reference standard results were used to select the individuals in the data sets. Therefore, we assumed that all the patients were evaluated with the reference standard before being evaluated with the index test. Under this assumption, it would be impossible to interpret the reference standard results with knowledge of the index test results.

Domain 4. Flow and Timing. Question: Did all patients receive a reference standard?

We assumed that all patients should have been selected using a reference standard.

Domain 4. Flow and Timing. Question: Did patients receive the same reference standard?

We assumed that all patients included in a study were selected using the same reference standard.

Domain 4. Flow and Timing. Question: Were all patients included in the analysis?

We assumed that all the patients selected into the study were included into the data sets in which the ML/AI intervention was applied.

Domain 4. Flow and Timing. Question: Could the patient flow have introduced bias?

We believe, due to the above assumptions, that the flow of the participants and the timing of the reference and index tests could not introduce bias into the study.

**Applicability concerns**

Domain 2. Index Test. Question: Is there concern that the index test, its conduct, or interpretation differ from the review question?

Due to the broad aim of our study to assess the available ML/AI interventions in PCOS, we believe that any ML/AI related index test would be applicable to the review question and therefore this question is irrelevant or our study.
