# Supplementary material for: Application of machine learning and artificial intelligence in the diagnosis and classification of polycystic ovarian syndrome: a systematic review
Source: Front Endocrinol (Lausanne). 2023 Sep 18;14:1106625. doi: 10.3389/fendo.2023.1106625 (PMC10542899; doi:10.3389/fendo.2023.1106625)
Supplement: Supplementary file 3 [file Table_3.docx]

**Supplementary Material 3. Full Search Strategy**

NIH Library Systematic Review Literature Search Documentation

Type of Review: Systematic

Research Question: To determine the use of artificial intelligence/ML in diagnosing PCOS

Databases Searched: PubMed, Embase, Cochrane Library, Web of Science, IEEE Xplore

| **Artificial Intelligence in Diagnosis of PCOS** | |
| --- | --- |
| Database: | Results |
| PubMed | 46 |
| Embase | 76 |
| Cochrane | 0 |
| Web of Science | 44 |
| IEEE Xplore Digital Library | 29 |
| TOTAL: (raw results) | 195 |
| **DUPLICATES REMOVED** | 60 |
| **Artificial Intelligence in Diagnosis of PCOS** | |
| Database: | Results |
| PubMed | 46 |
| Embase | 46 |
| Cochrane | 0 |
| Web of Science | 23 |
| IEEE Xplore Digital Library | 20 |
| TOTAL: (after duplicates removed) | 135 |

# Search Strategies:

**Database:** PubMed/MEDLINE

**Vendor:** US National Library of Medicine

**Date of Search:** Sept 7, 2021

**Publication Date Limits:** no limits
**Language Limits**: none applied

**Notes:** Use Advanced Search. Search keywords in the title and abstract fields, and use medical subject headings (MESH) controlled vocabulary for terms when available.

#1 - (Polycystic Ovary Syndrome[mh] OR "Polycystic Ovarian Syndrome"[tiab] OR "polycystic ovary syndrome"[tiab] OR "Polycystic Ovary Syndrome 1"[tiab] OR "Sclerocystic Ovarian Degeneration"[tiab] OR "Sclerocystic Ovaries"[tiab] OR "Sclerocystic Ovary Syndrome"[tiab] OR "Stein-Leventhal Syndrome"[tiab])

#2 - (machine learning[mh] OR artificial intelligence[mh] OR "artificial intelligence"[tiab] OR "neural network*"[tiab] OR "artificial neural network"[tiab] OR "bayesian network"[tiab] OR "binary mixed model classification"[tiab] OR "binary mixed model regression"[tiab] OR "tree cluster analysis"[tiab] OR "computer aid"[tiab] OR "convolutional neural network"[tiab] OR "data mining"[tiab] OR "deep learning"[tiab] OR "image recognition"[tiab] OR "machine learning"[tiab] OR "machine intelligence"[tiab] OR "natural language processing"[tiab] OR "pattern recognition"[tiab] OR "recurrent neural network*"[tiab] OR "reinforcement learning"[tiab] OR "support vector machine*"[tiab] OR "supervised learning"[tiab] OR "unsupervised learning"[tiab] OR "supervised machine learning"[tiab] OR "unsupervised machine learning"[tiab])

#1 AND #2

**Database:** Embase

**Vendor:** Elsevier

**Date of search:** Sept 9, 2021

**Publication Date limits**: none
**Language Limits:** none applied

**Notes:** Use Advanced Search. Limit keywords to the title and abstract fields, and use the EMTREE controlled vocabulary (EMTREE) terms.

**PCOS**

#1 - ('ovary polycystic disease'/exp OR "polycystic ovary disease":ti,ab OR "Polycystic Ovarian Syndrome":ti,ab OR "Polycystic Ovary Syndrome 1":ti,ab OR 'polycystic ovary syndrome*':ti,ab OR "Sclerocystic Ovarian Degeneration":ti,ab OR "Sclerocystic Ovaries":ti,ab OR "Sclerocystic Ovary Syndrome":ti,ab OR "Stein-Leventhal Syndrome":ti,ab)

**Artificial Intelligence**

#2 - ('artificial intelligence'/exp OR 'machine learning'/exp OR "artificial intelligence":ti,ab OR 'neural network*':ti,ab OR "artificial neural network":ti,ab OR "bayesian network":ti,ab OR "binary mixed model classification":ti,ab OR "binary mixed model regression":ti,ab OR "tree cluster analysis":ti,ab OR "computer aid":ti,ab OR "convolutional neural network":ti,ab OR "data mining":ti,ab OR "deep learning":ti,ab OR "image recognition":ti,ab OR "machine learning":ti,ab OR "machine intelligence":ti,ab OR "natural language processing":ti,ab OR "pattern recognition":ti,ab OR "recurrent neural network*":ti,ab OR "reinforcement learning":ti,ab OR "support vector machine*":ti,ab OR "supervised learning":ti,ab OR "unsupervised learning":ti,ab OR "supervised machine learning":ti,ab OR "unsupervised machine learning":ti,ab)

#1 AND #2

**Database:** Web of Science Core Collection -

Indexes=SCI-EXPANDED, SSCI, CPCI-S, CPCI-SSH, BKCI-S, BKCI-SSH, ESCI, CCR-EXPANDED, IC Timespan=All years

**Vendor:** Clarivate

**Date of search:** Sept 7, 2021

**Publication Date Limits:** No limits applied
**Language Limits:** none applied

**Notes:** Use Advanced Search. No controlled vocabulary, search using Topic field tag (TS) for keywords/phrases.

#1 - TS=("Polycystic Ovary Syndrome" OR "Polycystic Ovarian Syndrome" OR "Polycystic Ovary Syndrome 1" OR "Sclerocystic Ovarian Degeneration" OR "Sclerocystic Ovaries" OR "Sclerocystic Ovary Syndrome" OR "Stein-Leventhal Syndrome")

**Artificial Intelligence**

#2 – TS=("machine learning" OR "artificial intelligence" OR "artificial intelligence" OR "artificial neural network" OR "bayesian network" OR "binary mixed model classification" OR "binary mixed model regression" OR "tree cluster analysis" OR "computer aid" OR "convolutional neural network" OR "data mining" OR "deep learning" OR "image recognition" OR "machine learning" OR "machine intelligence" OR "natural language processing" OR "pattern recognition" OR "recurrent neural network*" OR "reinforcement learning" OR "support vector machine*" OR "supervised learning" OR "unsupervised learning" OR "supervised machine learning")

#1 AND #2

**Database:** Cochrane Library

**Vendor:** Wiley

**Date(s) Searched:** Sept 7, 2021
**Date range searched**: no limits
**Language Limits:** none applied

**Notes:** Use Search Manager. Search available MESH headings from PubMed in Cochrane, keywords/phrases in the title and the abstract.

#1 - [mh "polycystic ovary syndrome"] OR ("Polycystic Ovarian Syndrome" OR "Polycystic Ovary Syndrome 1" OR "polycystic ovary syndrome" OR "Sclerocystic Ovarian Degeneration" OR "Sclerocystic Ovaries" OR "Sclerocystic Ovary Syndrome" OR "Stein-Leventhal Syndrome"):ti,ab

#2 - [mh "machine learning"] OR [mh "artificial intelligence"] OR ("artificial intelligence" OR "artificial neural network" OR "bayesian network" OR "binary mixed model classification" OR "binary mixed model regression" OR "tree cluster analysis" OR "computer aid" OR "convolutional neural network" OR "data mining" OR "deep learning" OR "image recognition" OR "machine learning" OR "machine intelligence" OR "natural language processing" OR "pattern recognition" OR "recurrent neural network*" OR "reinforcement learning" OR "support vector machine*" OR "supervised learning" OR "unsupervised learning"):ti,ab

#1 AND #2

**Database:** IEEE Xplore Digital Library

**Vendor:** IEEE

**Date(s) Searched**: Sept 9, 2021
**Publication Date limits:** no date limits applied

#1 - ("Polycystic Ovary Syndrome" OR "Polycystic Ovarian Syndrome" OR "Polycystic Ovary Syndrome 1" OR "Sclerocystic Ovarian Degeneration" OR "Sclerocystic Ovaries" OR "Sclerocystic Ovary Syndrome" OR "Stein-Leventhal Syndrome")

#2 – ("machine learning" OR "artificial intelligence" OR "artificial intelligence" OR "artificial neural network" OR "bayesian network" OR "binary mixed model classification" OR "binary mixed model regression" OR "tree cluster analysis" OR "computer aid" OR "convolutional neural network" OR "data mining" OR "deep learning" OR "image recognition" OR "machine learning" OR "machine intelligence" OR "natural language processing" OR "pattern recognition" OR "recurrent neural network*" OR "reinforcement learning" OR "support vector machine*" OR "supervised learning" OR "unsupervised learning" OR "supervised machine learning")

#1 AND #2
